# “You Should Praise” – “You Should Kill”: The Contingent Negative Variation Indicates Moral Goodness and Badness

**DOI:** 10.3389/fnhum.2019.00432

**Published:** 2019-12-17

**Authors:** Christiane Neuhaus

**Affiliations:** Institute of Systematic Musicology, University of Hamburg, Hamburg, Germany

**Keywords:** CNV, motor-related negativity, N400, moral and immoral verbs, moral imperative, daily commands (“Ready–Set–Go”)

## Abstract

Moral advice (how to behave in life) is often conveyed by short, simple sentence constructions: “*You* – *should* – (plus verb with moral meaning).” Yet how moral prescriptions are processed has never been studied from a neurocognitive perspective. The results of this study suggest that the contingent negative variation (CNV) serves as a neural correlate for moral (and immoral) predictive phrases. In step 1, the original CNV paradigm (S1–S2–motor response) was extended using action-demanding *three-word* phrases taken from everyday contexts (e.g., “Ready–Set–Go”). In step 2, these commands were replaced by abstract words, each phrase then including a verb of moral or immoral meaning (e.g., “You should hope,” “You should praise,” and “You should lie”). During recording, each phrase type (e.g., moral or immoral) was presented blockwise. The task varied according to block order: Participants (*n* = 19) had to either listen attentively or respond with a finger tap immediately after the final word of a phrase. Electroencephalogram (EEG) data were bandpass filtered (0.1–30 Hz) and analyzed at the onset of the second word, yielding two independent responses: a bilateral CNV and a bilateral motor-related negativity, both decreasing from anterior to posterior. The results show that the CNV is sensitive to phrase constructions of moral resp. immoral valence. Thus, transfer to remote semantic fields seems possible. Importantly, this transfer is combined with a change of time frames, from restricted and highly pragmatic (as in the original paradigm) to indefinite and vague. Thus, a CNV may indicate not only preparation to action but also general guidelines for social life. An N400 occurring as an additional, task-dependent result cannot be sufficiently explained on the basis of the present data.

## Introduction

Doing good or evil, acting right or wrong? Human behavior is often guided by a set of social standards and a person’s value system. Ethical theory goes back to the 18th century and even further to antiquity; however, the neural mechanisms underlying moral behavior have only been studied over the past 20 years.

Morality may be considered as a complex product of beliefs and values. Almost every human process – be it cognitive, emotional, judgmental, or behavioral – may, in principle, be influenced by moral ideas. Handling moral issues requires a network of cortical and subcortical structures, which also raises the question of whether morality as such is embodied. Besides that, it is still unclear how parts of the moral brain are connected in detail (Casebeer, 2003; Moll et al., 2005; Park and Friston, 2013; Pascual et al., 2013). A common approach to study the brain mechanisms of moral decision making and moral behavior is to create a case of moral conflict, that is, “moral dilemma.” The term describes a paradoxical but real-life situation in which a person has to make a choice between two mutually exclusive options, which means, for example, that the person’s willingness to help one side is unavoidably combined with an inability to help the other. This catch-22 may lead to inner conflict, in particular in situations in which people need to be rescued from danger (e.g., Christensen and Gomila, 2012).

Processing *moral advice* is a different matter: Moral advice is language based. In its *simplest* form, it is conveyed by a special type of phrase construction: An addressing pronoun (“You”) is combined with a modal verb (“shall,” “should,” or “ought to”) followed by a verb with moral meaning (e.g., “praise” or “honor”). Moral phrases such as “*You should praise*” or “*You should honor*” are prescriptive in character and do not imply immediate, goal-directed forms of action.

From a philosophical point of view, Kant (1785, 1788) distinguishes between two types of moral sentence constructions, the *Categorical Imperative* on the one hand and the *Hypothetical Imperative* on the other – the first is absolute, that is, of general nature, expressing pure duty (e.g., to obey the law), the second occurs as an “if–then” construction pursuing a particular aim or purpose (e.g., if one wants to stay fit and healthy, one should do sports). Note that in its original wording, the *Categorical Imperative* (Kant, 1785) aims at a person’s right attitude (or “*good will*”), that is, the *a priori* before initiating or preparing an action (Ludwig, 1996).

“… Handle nur nach derjenigen Maxime, durch die du zugleich wollen kannst, daß sie ein allgemeines Gesetz werde.” (Kant, 1785 Grundlegung zur Metaphysik der Sitten, 1785)

(transl. as)

“… Act only according to that maxim whereby you can, at the same time, will that it should become an universal law.” (Kant, 1785. *The Groundwork of the Metaphysics of* Morals, 1785)

However, in the following study, the basic attitude of all participants toward stimuli and tasks was *a priori neutral*, that is, neither good nor bad. Strictly speaking, the precondition for a *Categorical Imperative* is not fulfilled. So the more general term “moral imperative” will be used instead.

Listening to a prescriptive “You-should”-phrase requires some preparatory mental state ahead of action, that is, not scheduled clearly within a precisely set time frame but still to be regarded as a sort of intention. Nonetheless, because moral prescriptions unfold sequentially, the focus of interest will be on the *time course* of processing., meaning from a methodological point of view that event-related potentials (ERPs) rather than functional MRI signals ought to be measured.

The aim of this study is to search for a neural correlate for simple predictive phrase constructions. For indicating this preparatory mental state, the *Contingent Negative Variation* (CNV, Walter, 1964) may be considered as a possible candidate.

The CNV is a particular type of anticipating potential, belonging to the class of *slow brain potentials* (slow waves). Anticipating potentials (as all types of slow waves) are clearly detectable negative voltage shifts. The main characteristics are late onset times (∼500 ms), a sustain part (plateau) of several hundred milliseconds, and amplitude maxima often larger than −10 μV.

The *CNV* appears bilaterally symmetrical with an amplitude maximum of approximately −20 μV and fronto-central topography (Tecce, 1972). However, the original CNV-eliciting paradigm – a simple two-part instruction – is extremely restrictive: A warning stimulus (e.g., a light flash) is followed by an imperative stimulus (e.g., a pure tone) that signals the subject to tap or press a key. In short: S1–S2 plus motor response. Whenever the time interval between S1 and S2 (offset–onset) exceeds 3 s, two sub-components, termed *O-wave* and *E-wave*, are clearly visible (e.g., Leuthold et al., 2004). The O-wave, distributed frontally, has its maximum still near the offset of S1, whereas the E-wave, located over the precentral/motor cortex, has its maximum in the proximity of S2. “O” stands for an unspecific orienting reaction, whereas the “E” may point either to expectancy or to a direct preparation of the motor response (e.g., Rohrbaugh et al., 1976; Mento, 2013). Further suggestions on the original type of underlying cognitive process are “event anticipation,” “attention,” “motivation” (for all, see Tecce, 1972), and “preparation to action in general” (Bareš et al., 2007). Other suggestions point toward “subjective time estimation,” provided that a paradigm with varying time lengths (offset S1–onset S2) is given (e.g., Elbert et al., 1991; Kononowicz and Penney, 2016).

In almost every study, clicks, tone bursts, and flashes have been used as stimuli, that is, basic types with ultrashort duration from visual and/or auditory modalities. Walter (1965) himself tried a modification by replacing clicks by words (carrying semantic meaning): “S1–S2” → “Ready – Now.”

At this point, I pose the following research questions:

Can this semantic approach be extended further?

(1) First, by using action-demanding *three-word* commands taken from everyday contexts? Examples are “Achtung–Fertig–Los” (“Ready–Set–Go”), which is a command used in sports, and “Drei–Zwei–Eins” (“Three–Two–One”), which is an action-demanding number sequence. This extended replication covers the first part of this study.
  Note that these types of phrase constructions clearly illustrate the meaning of the word “*Contingent*” (in *CNV*), which is “depending on a preceding item while also showing coherence without syntax.”
(2) Second, do *predictive phrases of either moral or immoral content* elicit a (primarily action-related) CNV? And if so, to what extent can a moral CNV be distinguished from its immoral counterpart?

This means, in a broader sense, that the original paradigm, using strictly set time markers (S1–S2), could also be applied to a *time-indefinite* frame.

In order to operationalize the second approach, the phrase constructions of part one (e.g., “Achtung–Fertig–Los”) (“Ready–Set–Go”) were replaced by abstract words, each phrase then including a verb of moral or of immoral meaning. Examples are “Du sollst beten” (“You should pray“), “Du sollst hoffen” (“You should hope”), or “Du sollst lügen” (“You should lie”). Note that during voice recording, the tone of the speaker intentionally conveyed a sense of command, so that any impression of sentence incompleteness, for instance, by a missing relative clause (“You should believe *[that]* ….”), could not arise.

According to my knowledge, only one language-based ERP study has been performed up to now to investigate the time-related responses of the “moral brain” (van Berkum et al., 2009). In that study, two groups with opposing views – religious-oriented persons on the one side and atheists on the other – were instructed to read 160 provoking statements of ethical content (e.g., about euthanasia or experiments with laboratory animals). Whenever sentences were inconsistent with a person’s value system, three ERP components – an early positivity, the N400, and a late positivity – significantly increased in amplitude, showing that the brain responds rapidly (within a time frame of 200 to 250 ms after word onset) whenever subjects become aware of value-based inconsistencies as a sort of expectancy violation.

Yet to my very best knowledge, no more than one early source does underpin the research question of the current study, which is “investigating whether or not the CNV indicates the valence resp. meaning of moral and immoral predictive three-word phrases.” This early source, published in 1897, is a short passage by James Henry Leuba, an American psychologist:

“… although psycho-physiological science is now in condition to provide the necessary data for a detailed psycho-physiology of the Moral Imperative, […] men […] have not directly addressed themselves to the consideration of this problem, and the […] Kantian metaphysical psychology of ethics has not yet been formally superseded by a psycho–physiology of the Moral Imperative in harmony with modern science.” (Leuba, 1897, p. 529)

Accordingly, the following hypotheses were tested:

(1) The CNV reacts to daily, action-demanding three-word commands.
(2) The CNV is a neural correlate of moral (and immoral) predictive phrase constructions.

## Materials and Methods

### Subjects

Nineteen undergraduate students, recruited from various disciplines, participated in this study (8 males and 11 females; average age = 23.7 years, *SD* = 5.04). Neither special expertise nor background knowledge was required to perform the task. According to self-report, each subject was right-handed and did not suffer from any neural disease or hearing loss. The study protocol was approved by the local ethics commission of the Faculty of Humanities of the University of Hamburg. Each participant gave written informed consent prior to investigation, and a course credit of one point was awarded.

### Stimuli

The stimulus material consisted of spoken three-word phrases. Two of (in total) four trial blocks included commands as part of everyday life; the other two presented moral resp. immoral predictive phrase constructions.

As daily commands, three phrase types were chosen:

(1) “Achtung–Fertig–Los” (“Ready–Set–Go”), (2) “Drei–Zwei–Eins” (“Three–Two–One”), and (3) “Ball–Kreis–Dreieck” (“Ball–Circle–Triangle”). Types 1 and 2 are overlearned (“contingent”) expressions, mainly used in a sporting context. Type 3 consisted of three unrelated nouns, each describing a geometric shape. Although these nouns belong to the same semantic field, word constellation *per se* does not call for action, making type 3 suitable for control.

With regard to the moral and immoral phrase constructions, the second experiment, a three-word *pattern*, was used: “You–should–(plus bi-syllable verb).” The verbs were taken from one out of three word classes: moral, immoral, and action for control (all in German) (see Table 1 for a complete list of verb expressions).

**TABLE 1:** List of (bi-syllable) verbs (plus translations) used in two of, in total, four blocks.

**Condition**					

**(MO)**		**(IMMO)**		**(ACT) (control condition)**	
		
**Moral** **verbs**	**Transl.**	**Immoral** **verbs**	**Transl.**	**Action** **verbs**	**Transl.**
Achten	Respect	Fluchen	Swear/curse	Angeln	Fish with rod
Beten	Pray	Foltern	Torture	Fischen	Catch fish
Bitten	Plead	Freveln	Sin	Gehen	Go/move
Danken	Thank	Heucheln	Feign	Golfen	Play golf
Ehren	Honor	Lästern	Knock	Hüpfen	Jump
Glauben	Believe	Lügen	Lie	Joggen	Jog
Heilen	Heal/cure	Metzeln	Slaughter	Klettern	Climb
Helfen	Help	Morden	Murder	Laufen	Walk/run
Hoffen	Hope	Quälen	Torment	Paddeln	Paddle
Loben	Praise	Schänden	Defile/ruin	Reiten	Ride
Opfern	Sacrifice	Schiessen	Shoot	Rudern	Row
Pflegen	Care for	Spotten	Mock	Schwimmen	Swim
Retten	Rescue	Stehlen	Steal	Tanzen	Dance
Schützen	Protect	Täuschen	Deceive	Tauchen	Dive
Spenden	Spend	Töten	Kill	Wandern	Hike

Regarding stimulus preparation, single words were spoken, recorded, and adjusted afterward according to acoustical standards, using professional equipment (Cubase 5 as software for audio recording; Audacity 2.0.6 for sound editing). The adjusted single words were then put together to the aforementioned three-word-phrases (saved in.wav format).

Across all categories and phrase examples, pause lengths were kept constant: between word 2 (offset) and word 3 (onset) the time interval was 550 ms through all conditions (cf. Tables 2a, 2b). Although a longer interval (offset 2 to onset 3) would have revealed possible sub-components (O- and E-waves) more clearly, an unnatural lengthening (e.g., of 1,000 ms) would have distracted the subject’s attention away from the original task, possibly leading to some additional bias. Tables 2a, 2b show word lengths and interstimulus intervals as used in the experiment.

**TABLE 2a:** Everyday phrases – word lengths and pauses (ms) (point of analysis: onset word 2).

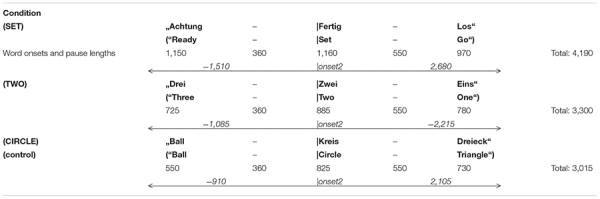

**TABLE 2b:** Phrases with moral and immoral meaning (plus control). Word lengths and pauses (ms) (point of analysis: onset word 2).

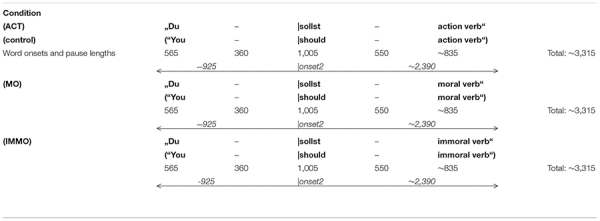

Trigger markers were set at every word onset (software GoldWave 6.24). However, as a point for analysis, only the marker at word onset 2 was taken.

Each condition consisted of 40 trials, resulting in 120 phrase examples per block: In accordance with Walter’s original paradigm (1964), the daily commands were strictly repeated 40 times (first experiment: 3 conditions × 40 trials), whereas the three-word *patterns* were repeated once or, partly, twice [second experiment: 3 conditions × (15 + 15 + 10 trials); repetitions within a block occurring in random order]. Note that because not many bi-syllable verbs of either moral or immoral meaning exist in the German language, repetition of some of the three-word *patterns* was necessary. This way, all participants heard all types of stimuli – the commands as part of everyday life as well as moral and immoral predictive phrase constructions (plus control) (Table 3 shows a detailed listing of blocks, tasks, and conditions).

**TABLE 3:** Block order, tasks, and conditions – overview.

**Block**	**Condition**	**Transl.**	**Task/Number of trials**
I	**Everyday commands (blockwise, exact repetition)**		Finger tap
	„Achtung–Fertig–Los“	[“Ready–Steady/GetSet–Go“]	40
	„Drei–Zwei–Eins“	[“Three–Two–One“]	40
	„Ball–Kreis–Dreieck“	[“Ball–Circle–Triangle“]	40
II	**Predictive phrases (blockwise)**		Finger tap
	„Du–sollst–[action verb]“	[“You–should–[action verb]“]	40
	„Du–sollst–[moral verb]“	[“You–should–[moral verb]“]	40
	„Du–sollst–[immoral verb]“	[“You–should–[immoral verb]“]	40
**Pause (∼10 min) to avoid carry-over effects**			
III	**Same as in I**		No tap, just listen
IV	**Same as in II**		No tap, just listen

### Task and Experimental Paradigm

The pure recording time was about 50 min. Each participant sat comfortably on a lounger in a dimmed and electrically shielded electroencephalogram (EEG) lab approximately 1.5 m in front of a monitor. Spoken phrases were binaurally presented via headphones (Sennheiser HD 203), and presentation flow was automatically maintained, using eevoke^TM^ as presentation software (version 3.1.5, ANT-Neuro, Netherlands). The experiment consisted of four blocks, each lasting approximately 10 min. Depending on the block number, participants were instructed to either listen attentively or tap with their right index finger on the button of a gamepad (Microsoft Xbox 360^TM^) immediately after the final word of a phrase was presented. Each trial started with a fixation cross (duration 1 s) to keep attention focused, then the respective auditory phrase example was played. Between trials, the interstimulus interval was 0.7 s.

Both types of blocks – the ones with daily commands and those with moral and immoral phrases – appeared twice, with and without tapping instruction (2 × 2 design). This way, each combination of task and stimulus type was equally frequent (cf. Table 3).

Note that because the focus of this study was on mental processes *ahead* of action, presentation mode for each stimulus class had to be blockwise. A blockwise design guaranteed that predictions about the next-appearing type of phrase could be made in a reliably and consistent manner, which is a necessary precondition for eliciting a CNV. A randomized form of stimulus presentation (as used in standard ERP designs) would have drawn attention to the only changing word within a phrase, that is, to *word onset 3*, probably eliciting cognitive processes *following*, but not *anticipating* the item. Having these aspects in mind, the decision was for a blockwise design.

In addition, block order was counterbalanced between subjects, starting with block I, II, III, or IV, that is, with pure listening or with tapping. This way, any bias toward or against a CNV due to sequential effects could be avoided. (Note that Table 3 shows only one of four options of block order.) The other types are as follows: II (listen)–I (listen)–IV (tap)–III (tap); III (tap)–IV (tap)–I (listen)–II (listen); IV (tap)–III (tap)–II (listen)–I (listen); each type was presented to a subgroup of five (resp. four) participants.

After half of the experiment, a short cartoon (duration 7 min) was shown for purposes of entertainment and distraction and also to avoid some sort of carryover effects between the session’s halves, that is, between blocks either requiring listening or tapping.

Each experimental run started with five trial examples to familiarize the participants with the task.

After the recording, subjects had to perform an additional rating test in which each moral and immoral verb was listed again in written form. The purpose was to let individuals evaluate in retrospect which verb he or she had processed in pure abstract form and which verb had evoked some motor associations during recording. (Listening to a moral “You should pray,” for example, could have evoked the motor association of “folding hands”). The idea behind was to distinguish between two processing types: a CNV evoked by motor images (in line with the theory of embodied cognition, e.g., Wilson and Foglia, 2017) and a CNV occurring for an abstract (non-image) processing style, or for verbs highly abstract in meaning (e.g., “You should lie” or “You should honor”). However, because this individual rating procedure did not yield a sufficient number of examples to build new subsets with noise-free brain responses, no decision could be made in favor of the first or second suggestion.

### EEG Recording

Each participant was asked to relax his or her facial muscles and keep head, neck, arms, and hands as motionless as possible. Eyes should be focused on the center of the monitor to reduce the number of eye blinks during recording (cf. general guidelines of ERP measurement, Picton et al., 2001).

Brain electrical activity was recorded with Ag/AgCl electrodes using a 32-channel electrode cap (waveguard^TM^) as well as Advanced Source Analysis (ASA^TM^, version 4.73) as recording software (both ANT-Neuro, Netherlands). Channel activity was referenced to the left mastoid (M1), and a position between FPz and Fz was used for the ground electrode. EEG signals were digitized with a sampling rate of 625 Hz during recording. In addition, ocular artifacts were registered with vertical and horizontal electrooculography (EOG) electrodes set above and below the right eye and at the outer canthi of both eyes, respectively. The impedance at each channel was kept below 10 kΩ. Tapping responses (button press) were also registered during recording.

### Data Analysis

#### Preprocessing

The preprocessing of EEG signals was done offline: First, raw data were filtered using an ASA-implemented bandpass filter of 0.1–30 Hz. The chosen cutoff frequencies of 0.1 and 30 Hz were in agreement with a range of standard filter settings to obtain a CNV (0.01–100 Hz, Bareš et al., 2007, also Macar and Vidal, 2003; 0.03–35 Hz, Trillenberg et al., 2000). Preprocessing was then continued, using eeprobe^TM^ as software (version 3.3.186, ANT-Neuro). EEG raw data were carefully examined for eye blinks, muscle activity, and technical artifacts in two steps: first, by applying an automatized program with amplitude thresholds of ±150 μV and second, by marking the residual artifacts carefully by hand. Only data sets with artifact-free trials were allowed for further averaging, resulting in approximately 27 trials per condition. Averaging was done for the following time ranges: −1,510 to 2,700 ms (condition “Ready–Set–Go”), −1,085 to 2,200 ms (“Three–Two–One”), −910 to 2,100 ms (“Ball–Circle–Triangle”). Note that the time ranges for averaging varied owing to different word resp. phrase lengths. However, duration was constant for blocks with predictive phrase types (moral, immoral, and action). Here, the time range for averaging was −925 to 2,300 ms throughout conditions.

Phrase constructions were analyzed at the onset of the second word. For baseline correction, a time interval of 100 ms pre-onset was used, and curves were finally subsumed to grand-average traces. To depict components clearly, an additional low-pass filter (8 Hz) was applied.

#### Statistical Analysis

Grand average results were first analyzed by visual inspection. Then, to validate the discernible components, eight 2-factor repeated measures analyses of variance (ANOVAs) were performed (within-subject design, statistical software package SPSS, version 25.0). The mean amplitude per time window and electrode served as the dependent variable. For statistical analysis with ANOVA, the following time ranges were chosen: (A) (validation of a CNV) 500–1,600 ms for block types I and III, consisting of daily commands with and without tapping instructions (2 ANOVAs), resp. 400–2,000 ms for block types II and IV, consisting of moral/immoral phrases with and without tapping instructions (2 ANOVAs); and (B) (validation of a motor-related negativity) 1,750–2,100 ms (block types I and III, 2 ANOVAs), resp. 2,000–2,100 ms (block types II and IV, 2 ANOVAs). The repeated measures factors are as follows: COND (condition, block types I and III; 3 levels): “Ready–|Set–Go,” “Three–|Two–One,” “Ball–|Circle–Triangle,” resp. COND (block types II and IV) “You–|should–action verb,” “You–|should–moral verb,” “You–|should–immoral verb”), and TOPO (topography, 4 levels, all block types): FRONT (fronto-central: F3, Fz, F4, FC1, FC2, C3, Cz, and C4), PARI (centro-parietal: CP1, CP2, CP5, CP6, P3, Pz, P4, and POz), LEFT (F3, F7, FC1, FC5, C3, CP1, CP5, and P3), and RIGHT (F4, F8, FC2, FC6, C4, CP2, CP6, and P4).

To prove to what extent a finger tap might influence the processing of conditions, also a three-factor (between blocks) repeated measures ANOVA was computed for each time window. By comparing block types I with III resp. II with IV, this ANOVA comprised the following factors: COND (3 levels), TOPO (4 levels), and (new) TASK (2 levels, finger tap vs. non-tap/pure listening) (Tables 4, 5 show the main statistical results).

**TABLE 4:** Results of a two-factor resp. three-factor repeated measures ANOVA **everyday commands**.

**2-factor**	**CNV**		**Motor-rel. negativity**		
**Analysis window (ms)**	**500–1,600**		**1,750–2,100**		
**Finger tap**					
COND	*F*_2,36_ = 17.04	*p* < 0.001	COND	*F*_2,36_ = 7.86	*p* = 0.001
Pairwise comparisons:			Pairwise comparisons:		
SET vs. TWO *p* = 0.001			SET vs. TWO *p* = 1.0 (n.s.)		
SET vs. CIRCLE *p* < 0.001			SET vs. CIRCLE *p* < 0.001		
TWO vs. CIRCLE *p* = 0.156 (n.s.)			TWO vs. CIRCLE *p* = 0.018		
TOPO	*F*_3,54_ = 1.34	*p* = 0.26 (n.s.)	TOPO	*F*_3,54_ = 8.92	*p* < 0.001
COND × TOPO	*F*_6,108_ = 5.07	*p* = 0.002	COND × TOPO	*F*_6,108_ = 7.77	*p* < 0.001
**No finger tap/just listen**					
COND	*F*_2,36_ = 5.69	*p* = 0.005	COND	*F*_2,36_ = 16.45	*p* < 0.001
Pairwise comparisons:			Pairwise comparisons:		
SET vs. TWO *p* = 1.0 (n.s.)			SET vs. TWO *p* = 0.017		
SET vs. CIRCLE *p* = 0.003			SET vs. CIRCLE *p* < 0.001		
TWO vs. CIRCLE *p* = 0.011			TWO vs. CIRCLE *p* = 0.025		
TOPO	*F*_3,54_ = 8.26	*p* < 0.001	TOPO	*F*_3,54_ = 2.19	*p* = 0.09 (n.s.)
COND × TOPO	*F*_6,108_ = 2.55	*p* = 0.028	COND × TOPO	*F*_6,108_ = 2.39	*p* = 0.046

**3-factor**	**CNV**		**Motor-rel. negativity**		
**Analysis window (ms)**	**500–1,600**		**1,750–2,100**		

TASK (only) (tap vs. no tap)	*F*_1,18_ = 0.41	*p* = 0.524 (n.s.)	TASK	*F*_1,18_ = 6.49	*p* = 0.012
COND × TASK	*F*_2,36_ = 10.05	*p* < 0.001	COND × TASK	*F*_2,36_ = 2.39	*p* = 0.097 (n.s.)
TOPO × TASK	*F*_3,54_ = 6.23	*p* < 0.001	TOPO × TASK	*F*_3,54_ = 8.95	*p* < 0.001
COND × TOPO × TASK	*F*_6,108_ = 2.13	*p* = 0.08 (n.s.)	COND × TOPO × TASK	*F*_6,108_ = 4.73	*p* = 0.001

**TABLE 5:** Results of a two-factor resp. three-factor repeated measures ANOVA **predictive moral and immoral phrases**.

**2-factor**	**CNV**		**Motor-rel. negativity**		
**Analysis window (ms)**	**400–2,000**		**2,000–2,100**		
**Finger tap**					
COND	*F*_2,36_ = 14.74	*p* < 0.001	COND	*F*_2,36_ = 20.08	*p* < 0.001
Pairwise comparisons:			Pairwise comparisons:		
ACT vs. MO *p* < 0.001			ACT vs. MO *p* < 0.001		
ACT vs. IMMO *p* < 0.001			ACT vs. IMMO *p* < 0.001		
MO vs. IMMO *p* = 0.461 (n.s.)			MO vs. IMMO *p* = 1.0 (n.s)		
TOPO	*F*_3,54_ = 5.72	*p* = 0.001	TOPO	*F*_3,54_ = 21.39	*p* < 0.001
COND × TOPO	*F*_6,108_ = 3.36	*p* = 0.023	COND × TOPO	*F*_6,108_ = 4.76	*p* = 0.003
**No finger tap/just listen**					
COND	*F*_2,36_ = 6.2	*p* = 0.004	COND	*F*_2,36_ = 0.68	*p* = 0.5 (n.s.)
Pairwise comparisons:			Pairwise comparisons:		
ACT vs. MO p = 1.0 (n.s.)			ACT vs. MO p = 1.0 (n.s.)		
ACT vs. IMMO *p* = 0.017			ACT vs. IMMO *p* = 1.0 (n.s)		
MO vs. IMMO *p* = 0.001			MO vs. IMMO *p* = 0.84 (n.s)		
TOPO	*F*_3,54_ = 2.34	*p* = 0.08 (n.s.)	TOPO	*F*_3,54_ = 2.42	*p* = 0.07 (n.s.)
COND × TOPO	*F*_6,108_ = 3.71	*p* = 0.004	COND × TOPO	*F*_6,108_ = 2.43	*p* = 0.032

**3-factor**	**CNV**		**Motor-rel. negativity**		
**Analysis window (ms)**	**400–2,000**		**2,000–2,100**		

TASK (only) (tap vs. no tap)	*F*_1,18_ = 10.41	*p* = 0.002	TASK	*F*_1,18_ = 42.11	*p* < 0.001
COND × TASK	*F*_2,36_ = 19.34	*p* < 0.001	COND × TASK	*F*_2,36_ = 14.99	*p* < 0.001
TOPO × TASK	*F*_3,54_ = 0.44	*p* = 0.72 (n.s.)	TOPO × TASK	*F*_3,54_ = 13.08	*p* < 0.001
COND × TOPO × TASK	*F*_6,108_ = 1.68	*p* = 0.16 (n.s.)	COND × TOPO × TASK	*F*_6,108_ = 2.78	*p* = 0.02

Degrees of freedom were corrected with Huynh and Feldt’s epsilon, and results were considered significant at the α-level of 0.05. To counteract the problem of multiple comparisons, Bonferroni was applied as a correction method for multiple testing.

## Results

### N1-P2 Components

**FIGURE 1:**
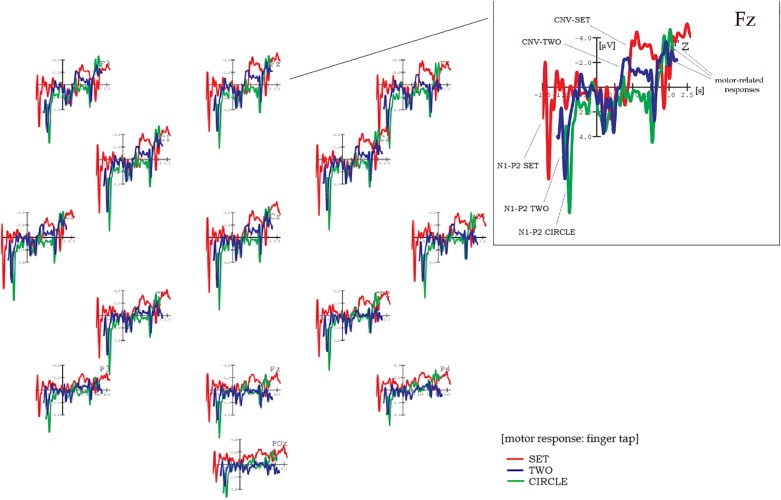
Grand average brain waves at word onset 2: responses to action-demanding daily commands followed by a finger tap. Red line: response to “***SET***” in: “Ready–***|Set***–Go” (orig. “Achtung–***|Fertig***–Los”); blue line: response to “***TWO***” in “Three–***|Two–***One” (orig. “Drei–***|Zwei–***Eins”); green line (control): response to “***CIRCLE***” in “Ball–***|Circle–***Triangle” (orig. “Ball–***|Kreis–***Dreieck”).

**FIGURE 2:**
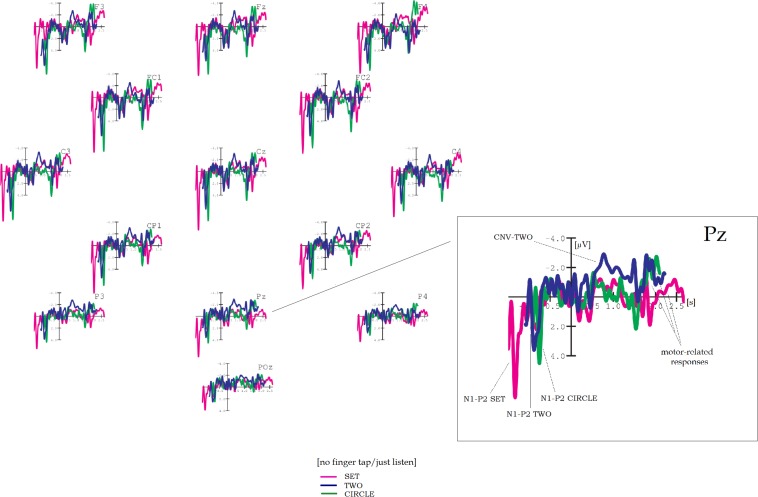
Grand average brain waves at word onset 2: responses to action-demanding daily commands for pure listening without finger tap. Pink line: response to “***SET***” in: “Ready–***|Set***–Go” (orig. “Achtung–***|Fertig***–Los”); blue line: response to “***TWO***” in “Three–***|Two–***One” (orig. “Drei–***|Zwei–***Eins”); green line (control): response to “***CIRCLE***” in “Ball–***|Circle–***Triangle” (orig. “Ball–***|Kreis–***Dreieck”).

**FIGURE 3:**
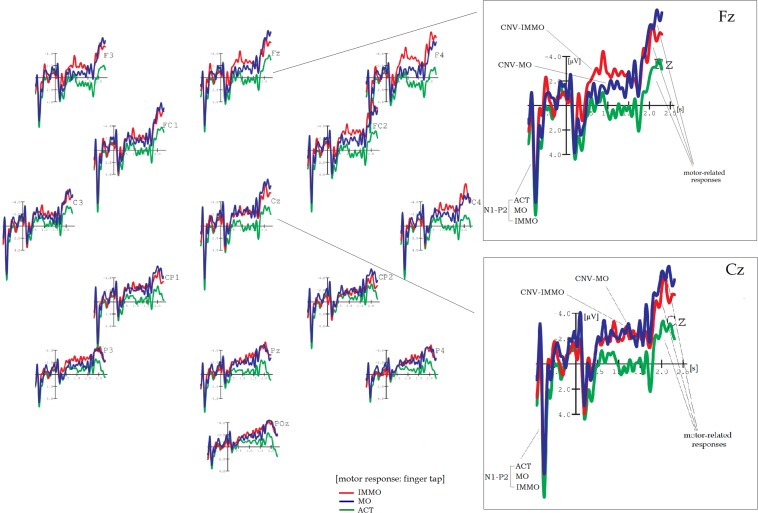
Grand average brain waves at word onset 2: responses to moral and immoral predictive phrases followed by a finger tap. Red line: response to “***should***” in “You–***|should–***immoral verb”; blue line: response to “***should***” in “You–***|should–***moral verb”; green line (control): response to “***should***” in “You–***|should–***action verb.”

**FIGURE 4:**
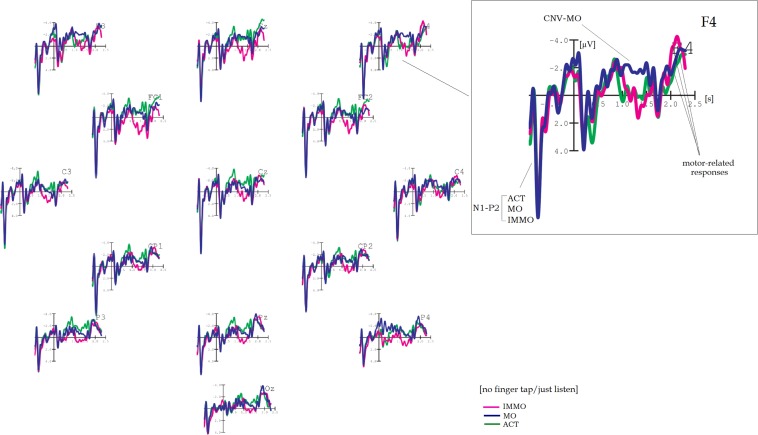
Grand average brain waves at word onset 2: responses to moral and immoral predictive phrases for pure listening without finger tap. Pink line: response to “***should***” in “You–***|should–***immoral verb”; blue line: response to “***should***” in “You–***|should–***moral verb”; green line (control): response to “***should***” in “You–***|should–***action verb.”

**FIGURE 5:**
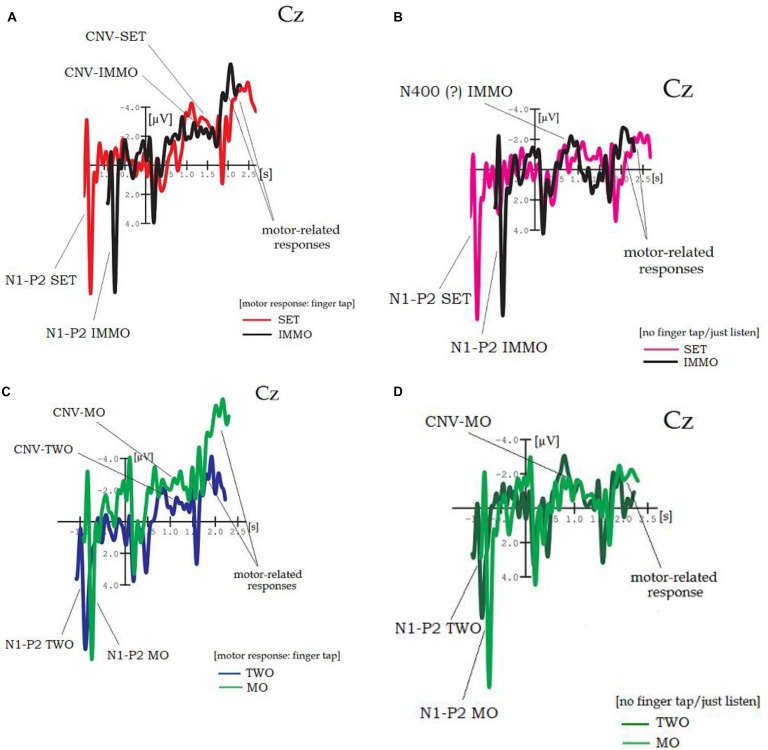
**(A–D)** Grand average brain waves at word onset 2, electrode Cz. Comparison across experiments: **(A,B) *SET vs. IMMO*** (with/without finger tap as instruction). Red/pink lines: response to “***SET***” in “Ready–***|Set***–Go” (orig. “Achtung–***|Fertig***–Los”), black lines: response to “***should***” in “You–***|should–***immoral verb.” **(C,D) *TWO vs. MO*** (with/without finger tap as instruction). Blue/dark green lines: response to “***TWO***” in “Three–***|Two–***One” (orig. “Drei–***|Zwei–***Eins”), green lines: response to “***should***” in “You–***|should–***moral verb.”

Grand average results are shown in Figures 1–5. Word onset 2 was chosen as the best-fitting point for analysis, marked by the zero point in each diagram. In all conditions, the brain’s reaction to word onset 2, the reference point, is indicated by a small N1-P2 (visible, but not labeled in the figures for reasons of clarity). Prior to that, a high-amplitude N1-P2 indicates the beginning of the entire phrase; however, owing to the chosen reference, it is located backward in the negative segments of the coordinate system, slightly compressed in form. Note that for daily commands, this initial N1-P2 showed a shift in latency, caused by different word lengths. Moral and immoral phrases, by contrast, were built according to pattern, thus, showing onset-consistency (cf. Figures 1, 2 vs. Figures 3, 4; for word lengths, see Tables 2a, 2b).

However, the main focus of the study is on how all phrase types are processed in detail. Thus, two further time ranges were defined: (A) 500–1,600 ms and (B) 1,750–2,100 ms for analyzing daily commands resp. (A) 400–2,000 ms and (B) 2,000–2,100 ms for analyzing moral and immoral phrases. (Note that these time windows [defined for visual inspection] are identical to those used for statistical analyses in SPSS).

### Action-Demanding Everyday Commands

#### Contingent Negative Variation

Figure 1 shows the brain results for action-demanding everyday commands followed by a finger tap as motor response. Target conditions are “Achtung–|Fertig–Los” (“Ready–Set–Go”) and “Drei–|Zwei–Eins” (“Three–Two–One”), whereas “Ball–|Kreis–Dreieck” (“Ball–Circle–Triangle”) served as control (hereinafter SET, TWO, and CIRCLE). SET and, partly, TWO reveal a clear CNV, most pronounced at fronto-central electrode positions, whereas CIRCLE, the (non-contingent) control condition, did not. A two-factor repeated measures ANOVA confirms these observations: a main effect COND (“Condition”) is highly significant (*F*_2_,_36_ = 17.04, *p* < 0.001), and pairwise comparisons attribute this to a significant amplitude difference between SET and TWO (*p* = 0.001), resp. SET and CIRCLE (*p* < 0.001; see Table 4 for more details). The first-order interaction COND × TOPO is also highly significant (*F*_6_,_108_ = 5.07, *p* = 0.002).

Detailed visual inspection reveals that CNV-SET and CNV-TWO differ in terms of shape and amplitude: Across electrodes, CNV-SET has its onset at 800 ms, a sustain part between 900 and 1,700 ms, and amplitude values between −2.4 and −4.1 μV. Furthermore, it appears with double-peak shape at centro-parietal positions (e.g., CP1 and CP2). Note that because no difference in topography can be found, one should be cautious to describe these (double-peak) sub-components as “O-” resp. “E-wave” (see e.g., Rohrbaugh et al., 1976; Leuthold et al., 2004). CNV-TWO – in contrast to CNV-SET – is less stable in shape and amplitude and more of peak than of plateau character. The amplitude maximum of −2.3 μV is at fronto-central electrode sites, and CNV-TWO almost diminishes from centro-parietal electrodes in the posterior direction.

Figure 2 shows results for the same phrase constellation, however, this time without finger tap as instruction (pure listening). For SET, no more than a slight tendency toward a small CNV can be observed, especially at fronto-central electrode sites of the right hemisphere (Fz, F4, FC6). The plateau is of 700-ms duration, and amplitude is of approximately −1.8 μV. This time, CNV-TWO is the dominant brain wave. It is peak shaped at fronto-central electrodes (amplitude maximum approximately −2.8 μV) and plateau shaped at parietal positions, mainly central and right-hemisphere electrodes (Pz, P4, and POz).

In spite of rather small amplitudes, ANOVA reveals a highly significant main effect for COND (*F*_2_,_36_ = 5.69, *p* = 0.005) caused by two highly significant pairwise comparisons, each with reference to the control (SET to CIRCLE, *p* = 0.003, and TWO to CIRCLE, *p* = 0.011). (Highly) significant results can also be found for TOPO (*F*_3_,_54_ = 8.26, *p* < 0.001) as well as for COND × TOPO (*F*_6_,_108_ = 2.55, *p* = 0.028).

In order to prove to what extent the CNV depends on the given task (finger tap vs. pure listening), an additional three-factor (between blocks) repeated measures ANOVA yielded two significant interactions with TASK as a factor (COND × TASK, *F*_2_,_36_ = 10.05, *p* < 0.001|TOPO × TASK, *F*_3_,_54_ = 6.23, *p* < 0.001, see Table 4).

#### Motor-Related Negativity

Within time window B (range from 1,750 to 2,100 ms), the results are as follows: In Figure 1, a steep ascending limb, simply called “*motor-related negativity*,” could be observed at fronto-central electrodes for all phrase conditions after finger tap, appearing immediately after the onset response to word 3. For SET, this motor-related negativity reaches amplitude values even higher than those for CIRCLE (e.g., at Cz), although slightly delayed in time due to varying lengths of the second word.

Through CIRCLE, the control condition, it can be demonstrated that this type of motor-related negativity is independent from the previous CNV: The central argument for independency is that for CIRCLE, a motor-related negativity is strongly pronounced, whereas a CNV is not existent (see Figure 1, fronto-central electrode sites).

Note that in search of an adequate expression, the term “readiness potential” (RP, original: “Bereitschaftspotential,” Kornhuber and Deecke, 1965) is certainly an initial suggestion: RP is another type of slow potential, well known for indicating the preparation of a voluntary motor response. On the other hand, motor responses as indicated by RP are rather unspecific, not taking any particular context or condition into account. More importantly, RP appears asymmetrically, indicating hand or finger movement for the contralateral side (see, e.g., Tecce, 1972 for a discussion). Because this sort of asymmetric development could not be found in the data here, I decided to skip this expression in favor of the neutral term “motor-related negativity.”

To validate the just described effects, again, eight 2-factor repeated measures ANOVAs were computed – now for a time range between 1,750 and 2,100 ms. For motor-related negativities after finger tap (Figure 1), a main effect of COND was highly significant (*F*_2_,_36_ = 7.86, *p* = 0.001), although visual inspection yielded something different. This apparent contradiction can partly be explained by inconsistent onset times caused by different word lengths, revealing steep and earlier-developing slopes for TWO resp. CIRCLE and a later-developing slope for SET (see Tables 2a, 2b, 3 for more details). Pairwise comparisons for each target condition related to the control (SET to CIRCLE and TWO to CIRCLE) yielded highly significant results: *p* < 0.001 and 0.018, respectively. In addition, amplitude decrease from anterior to posterior led to a highly significant (first-order) interaction (COND × TOPO, *F*_6_,_108_ = 7.77, *p* < 0.001).

Figure 2 shows a smaller motor-related negativity for the non-tap setting, although less homogenous between conditions.

A main effect of TASK and two interactions with TOPO were highly significant: TASK (*F*_1_,_18_ = 6.49, *p* < 0.012), TOPO × TASK (*F*_3_,_54_ = 8.95, *p* < 0.001), and COND × TOPO × TASK (*F*_6_,_108_ = 4.73, *p* = 0.001) (see Table 4).

### Moral and Immoral Predictive Phrases

#### Contingent Negative Variation

Figure 3 shows brain results for ACT, MO, and IMMO, and with finger tap as instruction. ACT stands for action phrases used for control, whereas MO, the moral condition, as well as IMMO, the immoral counterpart, consisted of predictive three-word phrases of either moral or immoral content. Again, word onset 2 served as the reference point for analysis through all conditions.

As visible in Figure 3, two CNVs occur while processing MO resp. IMMO, both starting 400 ms post-onset (word 2) and lasting approximately 1.1 s. Note that at frontal positions they appear in “layers” but show congruence at centro-parietal electrodes: Going into further details, CNV-IMMO, the uppermost “layer,” reaches amplitude values between −3 and −4.4 μV at Fz and F4, respectively, but is almost reduced to half at CP2 and Pz. For CNV-MO, on the contrary, amplitude was slightly increased at Cz and CP1 (showing a maximum of approximately −2.5 μV). Brain curves in response to ACT can be found loosely wrapped around the *x*-axis. ANOVA yields a highly significant main effect for COND (*F*_2_,_36_ = 14.74, *p* < 0.001), and, of course, those pairwise comparisons are highly significant in which ACT is the reference (MO–ACT, *p* < 0.001; IMMO–ACT, *p* < 0.001). Regarding topography, tendencies toward an anterior-to-posterior decrease in amplitude are confirmed by a significant (first-order) interaction COND × TOPO (*F*_6_,_108_ = 3.36, *p* = 0.023).

Figure 4 shows the grand average results for the non-tap counterparts: across electrode positions, CNV-IMMO is seriously reduced in amplitude (approximately −2 μV), now of peak shape, whereas CNV-MO preserves its shape fronto-centrally during a time interval of approximately 800 ms. This sustain part (plateau) makes the pairwise comparison between MO and IMMO highly significant (MO–IMMO, *p* = 0.001), also resulting in a highly significant main effect of COND (*F*_2_,_36_ = 6.2, *p* = 0.004), same for COND × TOPO (*F*_6_,_108_ = 3.71, *p* = 0.004).

Again, a three-factor repeated measures ANOVA confirms that the respective type of instruction, either tap or no tap/just listen, has a tremendous impact – this time on the development of CNV-IMMO (main effect of TASK, *F*_1_,_18_ = 10.41, *p* = 0.002, first-order interaction COND × TASK, *F*_2_,_36_ = 19.34, *p* < 0.001, see Table 5 for further results).

### Motor-Related Negativity

Motor-related negativities for MO and IMMO often appear twice as high in the tapping as in the non-tapping/just listen condition (cf. Figures 3, 4). Once more, the effect of TASK is confirmed by significant results of a three-factor (between blocks) repeated measures ANOVA (main effect of TASK, *F*_1_,_18_ = 42.11, *p* < 0.001, interaction COND × TASK, *F*_2_,_36_ = 14.99, *p* < 0.001).

Figure 3 shows (with finger tap as instruction) that motor-related negativities for MO and IMMO can clearly be distinguished from those for ACT, both reaching amplitude values between −6 and −7.7 μV in comparison with −3.5 μV for ACT fronto-centrally; however, at centro-parietal and parietal positions, all brain waves are sharply reduced in size. Pairwise comparisons between each target condition in relation to ACT (the control) reveal highly significant values (MO–ACT, *p* < 0.001; IMMO–ACT, *p* < 0.001; see Table 5 for more details).

For the non-tap instruction, a similar arrangement cannot be detected (Figure 4). This time amplitude height is approximately −3.5 μV across all conditions, also maintained in posterior direction. Only the first-order interaction was significant (COND × TOPO *F*_6_,_108_ = 2.43, *p* = 0.032).

Further insights can be gained by *comparison across experiments*, that is, SET vs. IMMO, resp. TWO vs. MO. The brain responses for these newly combined conditions are depicted in Figures 5A–D (each time at electrode Cz) with and without finger tap as instruction (again, the point for analysis is word onset 2). Figure 5A shows some striking similarities in shape and amplitude between CNV-SET and CNV-IMMO for the tapping instruction. Note that shifts in peak latency are caused by different word lengths resp. onset times across conditions. Interestingly, IMMO in Figure 5B (condition “no-tap/just listen”) does not develop a sustain part (plateau) between 500 and 1,600 ms, neither at Cz nor at any other electrode position (not depicted here). This, however, is usually regarded as a specific morphological attribute of the CNV. Instead, Figure 5B reveals a component-like shape (due to a rapidly decreasing slope), possibly indicating an N400-IMMO, although peak latency (800 ms post-onset) is somehow late. Thus, Figure 5B may be considered as an exception.

### Tapping Results

The time points for tapping with the index finger (averaged over all participants) are listed in Table 6, separately for block and condition. Subjects pressed the button of the gamepad more or less with constant speed depending on their subjective impression of phrase completion. Note that the total length for each phrase construction is in fact slightly longer because for this, the decay of the final word has also been taken into account (as listed in Tables 2a, 2b). Another point is that button press responses, as recorded with eevoke^TM^, use the *onset* of the *entire phrase*, that is, the very first cue point, as a basis for computation. In regard to button press responses alone, the final word, either onset or offset, would certainly have been a more appropriate reference point in accordance with the given instruction.

**TABLE 6:** Time points for tapping with index finger (instruction in two of four blocks).

**Condition**	**SET**	**TWO**	**CIRCLE**	**ACT**	**MO**	**IMMO**
Mean	4, 014.16	3, 232.12	3, 096.13	3, 262.82	3, 298.14	3, 425.34
*SD*	295.08	250.66	121.07	155.43	137.01	165.61

**M* and *SD* over all subjects (*n* = 19) (ms).*

## Discussion

The present study aimed at testing whether or not the CNV serves as a neural correlate for moral and immoral predictive phrase constructions.

The overall idea of this study consisted of testing three-word phrases that did or did not call for an action. In a first step, the original CNV paradigm (S1–S2–motor response, Walter, 1964) was extended by replacing clicks and light flashes (of ultrashort duration) with action-demanding daily commands. The second level was abstract, investigating whether or not the CNV might react to a predictive “You–should” followed by either a verb of moral or of immoral meaning. In the latter case, the CNV might be considered as a neural correlate of the moral imperative, also implying that the original paradigm with strictly set time markers (S1–S2) could be applied to situations in which time constraints are less strict.

In general, both research questions can be answered into the positive, showing that the range of application is broader than previously thought.

### Action-Demanding Everyday Phrases

Action-demanding daily commands (followed by a finger tap) revealed a clear CNV, most evident for SET (see Figure 1). As a similar CNV for CIRCLE, consisting of unrelated word items (control condition), failed to appear, the *contingency of words* might be a necessary prerequisite for eliciting a CNV. Note that whenever contingency is given, expressions are overlearned as in “Achtung–Fertig–Los” (“Ready–Set–Go”); that is, words are not selected by chance (as in the control). This “contingency CNV” thus can give supplemental information about a certain state of the brain in action while preparing, for example, a finger tap, a sprint, or a long jump. In a wider context, contingency has also been recognized as having a key role in aphasia research: van Lancker Sidtis and Postman (2006), for instance, could show that speech formulas such as “All right,” “Thank you,” and “How are you?” are well preserved in patients with aphasia and can better be trained than spontaneous, non-overlearned expressions (see also Stahl and Kotz, 2014).

An entirely different matter is to compare results between blocks that have TASK as the only distinction (I vs. III, resp. II vs. IV). In general, the type of instruction (“active finger tap” vs. “passive non-tap/just listen”) has a tremendous impact on the development of a CNV (cf. Figure 1 vs. Figure 2 and Figure 3 vs. Figure 4): In blocks including a finger tap as instruction (I and II), a CNV can be found in both target conditions (SET, TWO, resp. MO, IMMO) but not in control conditions (CIRCLE resp. ACT), showing that it precisely reacts to content resp. the valence of verbs. For blocks including a passive “just listen” instruction (i.e., III and IV), the situation is less clear. From this, I conclude that a finger tap (or other motor response) is a fundamental precondition for any type of CNV design to fulfill the formal criterion of the CNV paradigm regardless of content. That is, *within* this standard setting (including finger tap), it still seems possible to distinguish between verb meanings (moral, immoral, and action) and between responses on a concrete vs. abstract level (cf. the study’s first and second parts).

In spite of that, TWO shows a slight plateau-shaped CNV at parietal, mainly right-hemisphere electrodes even for the non-tap instruction (Pz, P4, and POz; see Figure 2). Because of parietal occurrence, this “passive” CNV for TWO (appearing in a non-tap condition) might indicate activation (resp. retrieval) of pure numeral information, which, as such, is stored in the intraparietal sulcus (IPS, see e.g., Nieder and Dehaene, 2009).

### Moral and Immoral Predictive Phrases

Figures 3, 4 reveal that, indeed, the brain reacts to predictive phrase constructions of moral and immoral contents. Thus, transfer to a remote semantic field seems possible, maybe owing to certain similarities in sequence structure between the original paradigm (S1–S2) and the three-word pattern (“You should [plus moral resp. immoral verb]”). Importantly, this transfer is combined with a change of time frames (or time standards), from “restricted” and “highly pragmatic” (S1–S2) to “indefinite” and “vague,” because guidelines for social life (“You should”) cannot be transmitted in a time-precise format. Figure 3 shows that whenever combined with a finger tap, a clearly visible CNV develops for both, the processing of *moral* as well as of *immoral* phrase types (in contrast to phrases with action verbs as control). CNV-IMMO decreases in amplitude in posterior direction, whereas CNV-MO remains more or less stable.

Let me first interpret results for CNV-IMMO alone. In my opinion, three explanations seem plausible; nonetheless, each can easily be refuted: first, its distinctive frontal appearance suggests that listening to immoral (“evil”) verbs combined with an instruction for execution (here, a finger tap) evokes a clash with a person’s value system (assumed to be stored in ventromedial prefrontal cortex [vmPFC], e.g., D’Argembeau, 2013), possibly eliciting resistance, that is, an inner unwillingness to comply with the tapping instruction. This may be indicated by a negativity, thus revealing strong but unsuccessful inhibition caused by a “forced” instruction to tap regardless of the verb’s content. This form of unsuccessful inhibition followed by execution has already been investigated by using a “stop–signal paradigm.” There, unsuccessful stopping was often indicated by an error-related negativity (ERN) combined with activation of medial frontal regions as well as of the pre-supplementary motor area (pre-SMA) and the anterior cingulate cortex (ACC) (Verbruggen and Logan, 2008). However, in the current study, this type of explanation may be questioned by CNV-MO as a response to processing moral phrases, because at some other (namely, centro-parietal) electrode sites, its appearance is very similar to that of CNV-IMMO.

Another suggestion on how to interpret results for CNV-IMMO alone goes in the direction of mental imagery: During recording, certain immoral (as well as moral) phrases such as “You should kill|steal|pray” could have lively been seen in the mind’s eye, possibly resulting in a clearly perceptible negativity. Note that in order to distinguish between a CNV evoked by motor associations and that occurring for an abstract (non-image) processing of verbs, an additional rating test was performed immediately after EEG recording. However, because judgments differed from subject to subject (making individual-based re-codings of trigger markers necessary), the number of evaluated examples per condition was not sufficient to perform additional sub-analyses. In spite of that, strong counterevidence is given by ACT, the control condition: Action phrases such as “You should swim|climb|paddle” are rather concrete and thus should have been imagined even more vividly. Anyhow, for ACT, the control, a CNV similar to that for IMMO resp. MO could not be detected, making this chain of argumentation somehow not plausible.

What about interpreting CNV-IMMO as a broadly shaped N400? Note that, at first glance, two criteria for an N400 seem to be fulfilled: First, a certain incongruity might exist between the immoral phrase types and the participants’ personal beliefs (cf. van Berkum et al., 2009). Second, the negative shift for IMMO occurs primarily at centro-parietal electrodes, pointing to sources typical for an N400 standard paradigm (e.g., van Petten et al., 1999). However, at centro-parietal electrodes, similar brain reactions are also evoked by the moral condition, showing that CNV-MO and CNV-IMMO are almost congruent (e.g., at Cz).

Having in mind that, in principle, an N400 either indicates violation of word expectancy (e.g., Kutas and Hillyard, 1980) or an inner conflict regarding personal values (e.g., van Berkum et al., 2009), at least one of these accepted opinions does not match with this observation of centro-parietal *congruence*. However, the similar responses to MO and IMMO at centro-parietal electrode sites may simply reflect that verbs in the moral condition were in some ways *as unexpected as* those in the IMMO condition. This might allow to draw conclusions about moral and immoral concepts as mental representations resp. part of the internalized repertoire in that obviously both, the verbs of moral and of immoral meaning, have to be considered as deviations in regard to subject-related vocabulary. From a methodological point of view, this observation of centro-parietal *congruence* is in good agreement with the choice of presentation mode (blockwise), yielding some sort of *overall expectancy* with regard to the respective verb class. On the other hand, certain words, especially those with immoral meaning (kill|torment|feign|deceive|slaughter), might cause non-predictable, individually different surprise, meaning that some unpredictable N400 effects might be traced back to the single-trial level, some unpredictable N400 effects could be possible. This issue needs further investigation to decide in favor of the first or second suggestion.

However, this centro-parietal congruence between CNV-MO and CNV-IMMO in Figure 3 makes one start to think of another point, which is a certain common source for processing “the good” and “the evil.” Note that from a higher perspective, this implies that “the evil” does not necessarily have greater power, resp. a stronger effect on the mind than “the good” (but see Baumeister et al., 2001, for a different point of view). In other words, in most situations, moral and immoral forces should be balanced in the brains of the majority of people.

Let me continue with Figure 4 showing brain results for a somehow “passive,” no-tap/just-listen-instruction. MO (with example phrases such as “You should honor|praise|thank”) is the only condition for which a fronto-centrally distributed CNV was slightly preserved in shape. According to this, a “passive” CNV (due to instruction) might indicate that MO-related cognitive processes are abstract, that is, less orientated toward action, meaning that internalized moral values (stored in vmPFC) could also be activated by pure listening, that is, without the necessity to act.

Furthermore, the comparison between Figures 5A,B (showing responses to IMMO with and without tapping instructions, either eliciting a CNV or an N400) raises the question of a *different processing mode depending on the given task*: That is, although action relatedness (the action mode) might predominate in the first case (IMMO plus motor response), it does not in the second (IMMO plus non-tap instruction), and this moment of reflection on the meaning (or moral valence) of the respective phrase – instead of action preparation – would help explain why this tendency toward a (potential) N400 can be observed. However, this CNV-N400 debate raised within the context of moral issues, obviously depending on task resp. instruction, cannot be sufficiently pursued on the basis of the present data.

## Conclusion

Taken together, the results of this study suggest that the CNV may serve as an indicator in new, and partly remote, semantic contexts: first, by indicating action relatedness on a concrete level (everyday life) and second, by reacting to verbs of moral resp. immoral valence on a highly abstract level.

However, results could be more precise by specifying “group” as a factor. In detail, participants studying theology may have different ideas on morality in comparison to subjects with borderline personality and a criminal record but equal intellectual capacities. Cima et al. (2010), for instance, argue that what makes psychopaths carry out criminal actions is not a lack of understanding of the moral rights and wrongs in general but rather a deficit in inhibition control.

Even so, one should further elaborate on the point *what specific type of process* a moral CNV might indicate: Is it a process based on motor images, that is, imagined mental simulations of actions, or is it a process indicating that moral verb-understanding is abstract? Regarding the first aspect, moral prescriptions would primarily activate the sensuo-motor system, which is in line with the theory of “embodied cognition” (e.g., Gallese and Lakoff, 2005; also Wilson and Foglia, 2017). (In my opinion, this is not necessarily in conflict with the assumption that moral values are prefrontally stored in vmPFC.) Furthermore, Jeannerod (2001) argues that whenever actions are purely imagined, motor areas are co-activated in a subliminal manner, meaning that representations of actions may exist in a certain motor format, which may help facilitate execution.

In regard to the second suggestion, that is, assuming that moral verbs ought to be understood in an abstract way, processing might tend toward disembodiment, that is, toward advice given “from outside” (by society or religion) (for a discussion, see Chatterjee, 2010). From a methodological point of view, a possible strategy to distinguish between different degrees of embodiment (or abstraction) while processing predictive phrases could be *phrase transformation* in that both will be replaced, the pronoun as well as the modal verb: from “**You should**” (e.g., “honor,” “pray,” or “lie”) to “**I should**” (“honor,” “pray,” or “lie”), further replaced by “**I will**” (“honor,” “pray,” or “lie”). This means that brain responses to moral and immoral phrase constructions – currently built from the *second-person perspective* (“You”) – might then be elicited by phrase constructions from a *first-person perspective* (“I”), through which, typically, *body-own* action concepts are transmitted.

## Ethics Statement

This study was carried out in accordance with the ethics guidelines of the University of Hamburg. All subjects gave written informed consent in accordance with the Declaration of Helsinki. The protocol was approved by the local ethics commission of the Faculty of Humanities of the University of Hamburg.

## Author Contributions

CN designed the study, performed the research, analyzed the data, and wrote the manuscript.

## Conflict of Interest

The author declares that the research was conducted in the absence of any commercial or financial relationships that could be construed as a potential conflict of interest.
